# Antegrade common bile duct (CBD) stenting after laparoscopic CBD exploration

**DOI:** 10.4103/0972-9941.30682

**Published:** 2007

**Authors:** Samik Kumar Bandyopadhyay, Shashi Khanna, Bimalendu Sen, Om Tantia

**Affiliations:** Department of Minimal Access Surgery, ILS Multispeciality Clinic, Salt Lake City, Kolkata, India

**Keywords:** Antegrade stenting, choledochoduodenostomy, choledocholithiasis, laparoscopic common bile duct exploration, T-tube drainage

## Abstract

Laparoscopic common bile duct exploration (LCBDE) has been found to be a safe, efficient and cost-effective treatment for choledocholithiasis. Following LCBDE, the clearance may be ascertained by a cholangiogram or choledochoscopy. The common bile duct (CBD) may be closed primarily with or without a stent *in situ* or may be drained by means of a T-tube or a biliary enteric anastomosis.

**Materials and Methods:**

In our series of 464 patients of choledocholithiasis, 100 patients underwent closure of the CBD with an indwelling antegrade stent following LCBDE. LCBDE was performed by direct massage of CBD, saline lavage, direct pickup with choledocholithotomy forceps or by basketing. Fragmentation of impacted stones *in situ* was performed in a few patients. Completion choledochoscopy was performed by means of a pediatric bronchoscope. A 10-cm, 7 Fr. double-flap biliary stent was placed *in situ* after LCBDE.

**Results:**

There was no mortality in the series. There was no conversion either. The median duration of the operation was 75 min. The mean postoperative hospital stay was 3.5 days. One patient had a minor postoperative biliary leak. One patient had a right sub-hepatic collection. Four patients developed postoperative port infection. The stents were removed endoscopically after 4 weeks. Sixty-eight patients could be followed up till 1 year. There has been no incidence of residual disease and the patients on follow-up are asymptomatic.

**Conclusion:**

In our experience, a single stage laparoscopic treatment of cholelithiasis with choledocholithiasis is a safe, viable and cost-effective option. Closure of the CBD over an antegrade stent is a feasible option but requires advanced skills in minimal access surgical techniques, especially endosuturing. The procedure may be performed safely in expert hands without mortality and with negligible morbidity.

Laparoscopic common bile duct exploration (LCBDE) has been found to be a safe, efficient and cost-effective treatment for choledocholithiasis.[1,2] Up to 16% of patients with gallstone disease may have concurrent choledocholithiasis.[3–5] In a conventional ‘open’ common bile duct (CBD) exploration, a clearance rate of more than 90% was accepted as a standard of care.[6] However, this implied that nearly 10% of these patients had a missed or a slipped stone and were liable to undergo re-exploration.

Endoscopic CBD clearance following an endoscopic sphincterotomy gained popularity as a means to tackle choledocholithiasis with cholelithiasis. The introduction of laparoscopy in treating choledocholithiasis as a single stage treatment raised an important issue - how to choose the treatment for each particular patient.

We started performing CBD explorations in 1997. As per our institutional protocol, we have used different modalities to clear the CBD. However, due to the limitations involved in transcystic LCBDE, we have always performed LCBDE through a properly performed choledochotomy. Hereby, we present our experience with antegrade stenting of the common bile duct following LCBDE.

## MATERIALS AND METHODS

A retrospective study of the patients treated for choledocholithiasis in the period between August 2000 and March 2005 was performed. The specially designed departmental software was utilized to retrieve the data. A single surgical team at our institute performed 5,043 laparoscopic cholecystectomies between August 2000 and March 2005. In the same period, 464 patients with choledocholithiasis were treated at the institute, of whom 22 patients had a primary or a slipped / missed CBD stone following cholecystectomy.

The series included 318 female and 116 male patients. The majority of the patients (210) belonged to the age group between 40 and 60 years, followed by the age groups 20-40 years (133) and 60-80 years (107). Only 14 patients were aged less than 14 years. The commonest presentation was abdominal pain, either in the epigastrium or the right hypochondrium (50.5%). A history of jaundice or icterus at presentation was found in 35.19% of the patients, whereas 21% of patients had a history of prior hospitalization due to acute pain in abdomen and a conservative management. There is a fixed institutional protocol that is implemented while treating patients with choledocholithiasis [Table 1].

**Table 1:** Institutional protocol for management of cholelithiasis with choledocholithiasis

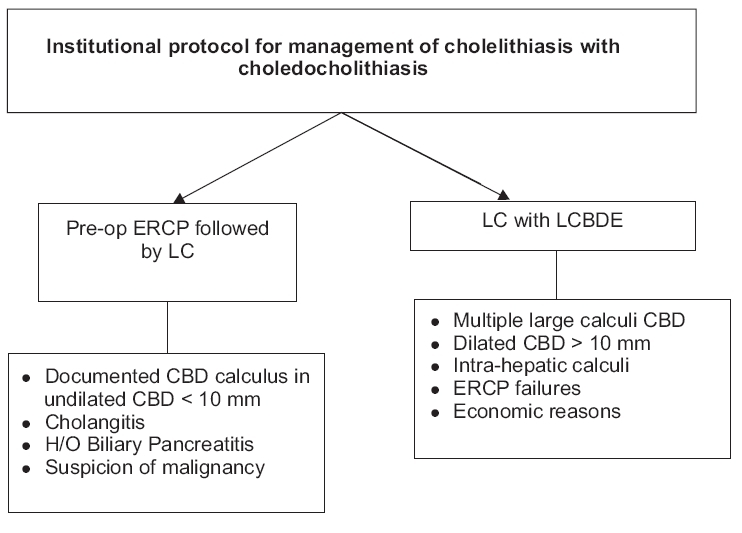

Out of 464 patients of confirmed choledocholithiasis, 316 patients were referred to endoscopic retrograde cholangiopancreatography (ERCP), of whom 262 patients underwent a successful clearance of CBD stones [Table 2]. Sixteen patients with a negative ERCP underwent laparoscopic cholecystectomy (LC) only. There were 38 ERCP failures due to various reasons [Table 2]. These patients subsequently underwent LCBDE. Of the 22 patients with post-cholecystectomy CBD stones, 7 were taken up for LCBDE directly; whereas 3 others who had a failed ERCP extraction also underwent LCBDE. As per the institutional protocol, patients with severe pancreatitis were initially treated conservatively and resuscitated before being subjected to any interventional operation. Patients with pancreatitis but otherwise stable (Ranson's score <4) were treated as per the institutional protocol.

**Table 2:** Break up endoscopic retrograde cholangiopancreatography group

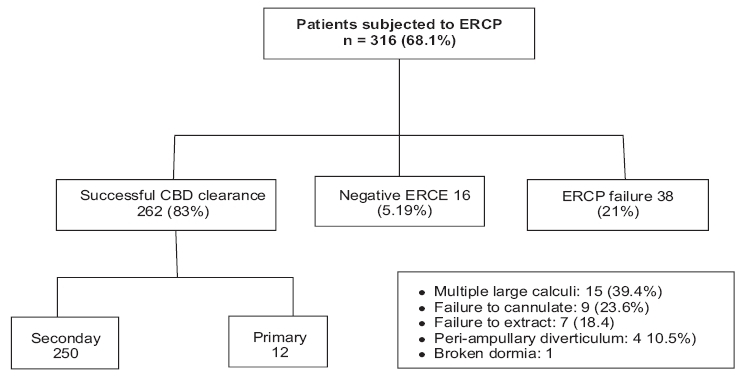

In patients with a strong suspicion of CBD stones on clinical and biochemical indicators, not otherwise documented by preoperative sonogram (in most of the cases) or magnetic resonance cholangiopancreatography (MRCP) (in a selected few), we prefer to perform a per-operative cholangiogram (POC). MRCP was done only in selected patients in our series due to financial constraints. In 69 patients who underwent POC, 52 patients were found to have a CBD stone and underwent subsequent LCBDE with LC.

LCBDE was performed in 181 patients, of which LC with LCBDE was done in 171 patients, while 10 patients of primary / missed CBD calculus underwent only LCBDE. Post-exploratory T-tube drainage of CBD was done in 18 patients, primary closure of CBD without any internal drainage was done in 4 patients while laparoscopic choledocho-enteric anastomosis was performed in 59 patients (choledochoduodenostomy - 52 and choledochojejunostomy - 7). One hundred patients had undergone post-exploratory antegrade biliary stenting with closure of CBD on the stent [Table 3]. Of the 18 patients with T-tube drainage, 1 patient had bouts of recurrent cholangitis and most patients were clearly unhappy at having a tube from their body. Patients from a rural setting were clearly unwilling to go home with a tube, resulting in their requiring a prolonged hospital stay. These feedbacks played an important role in offering the patients the benefit of stented choledochorrhaphy.

**Table 3:** Laparoscopic common bile duct exploration flow chart

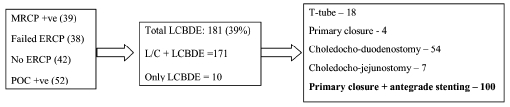

A standard 4-port approach was utilized. An optional fifth port (5 mm) was introduced midway between the right hypochondrial and umbilical ports to allow the introduction of a catheter for lavage or suction of the CBD.

A standard cholecystectomy was performed laparoscopically, but the cystic duct was not divided and also the gallbladder was not lifted from the liver bed. Mirizzi syndrome necessitating transaction of the gallbladder at the neck was encountered in nine patients. The remnant cuff was subsequently sutured with 3-0 vicryl sutures. Cholecysto-enteric fistula was found in five patients (cholecystoduodenal - 4, cholecystocolic - 1). The fistulas were isolated during operation using a combination of sharp and blunt dissection and fistulectomy was performed with scissors. The ensuing rent in the bowel was closed with interrupted 3-0 vicryl sutures in two layers.

Per-operative cholangiogram was performed in 69 patients. In these patients, following the skeletonization of the cystic duct, a medium large clip (Ligaclip LT 300; Ethicon Endosurgery Inc, Cincinnati, USA) was placed towards the gallbladder and a nick was created on the cystic duct (CD), which was then cannulated with a 4-5 Fr. ureteric catheter (which had been pre-primed with normal saline to expel possible air bubbles). A clip was then applied on the CD to prevent leakage of contrast material. Ports were withdrawn, the abdomen was deflated and a cholangiogram was obtained with a Digital C arm Fluoroscopy (real time imaging). If a radiolucent shadow was noted, the position of the patient was altered to rule out an air bubble and the entry of contrast material into the duodenum was noted. On the basis of a positive POC, 52 patients underwent simultaneous LCBDE with LC.

The duodenum was then kocherized up to the lateral border of the inferior vena cava to straighten out the CBD. The position of the CBD was confirmed by needle aspiration with a 24 G needle. The CBD was then opened longitudinally between 3-0 silk. We have avoided transcystic LCBDE because of its inherent limitations [Table 4].

**Table 4:** Limitations, contraindications and complications of trans-cystic laparoscopic common bile duct exploration[7]

Limitations:
Small cystic duct diameter
Low common bile duct cystic duct junction
Obstructive valves in cystic duct
Contraindications:
Large multiple stones in common bile duct
Stones in upper common bile duct
Complications
Common bile duct perforation
Common bile duct avulsion

**Figure 1:**
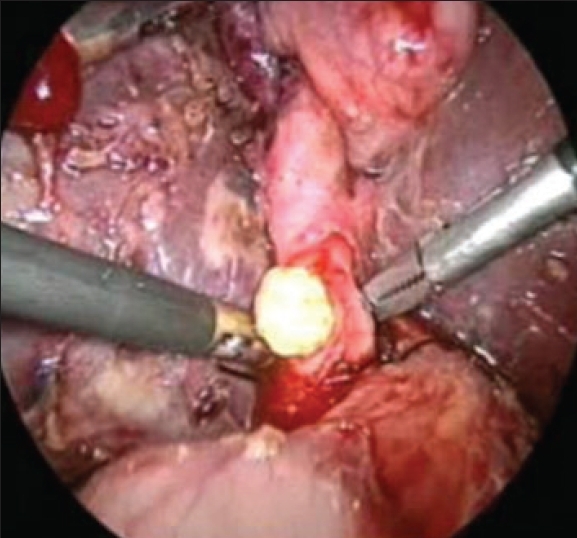
Stone being delivered from CBD by gentle milking

**Figure 2:**
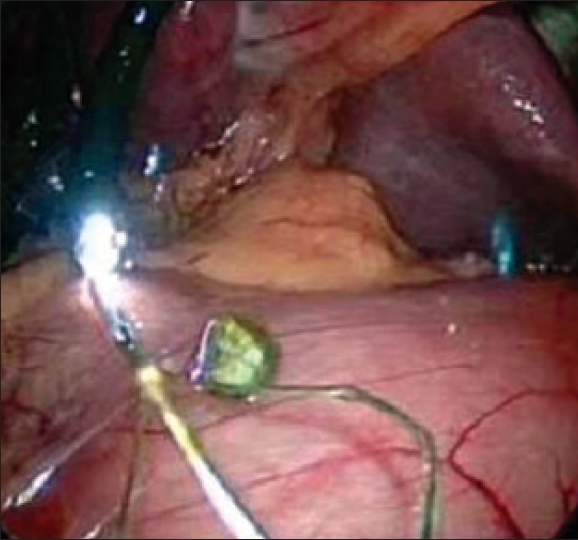
Stone being delivered from CBD by a Dormia basket

In most of the patients, the stones were recovered by spontaneous extrusion on choledochotomy or by gentle milking with the instruments [Figure 1]. A 12 Fr. Ryles tube (with its tip cut off) introduced through the accessory 5-mm port was used to perform proximal and distal lavage of the common bile duct and help in extrusion of the stones. Stones were also removed by means of a Dormia basket [Figure 2] or a Fogarty catheter under direct flexible choledochoscopic control. In a few patients, curved Desjardines choledocholithotomy forceps were introduced directly through the epigastric wound after removal of the 10-mm epigastric port, in order to clear the distal CBD.

In patients with impacted stones in the CBD or common hepatic duct, a rigid ureteroscope introduced through an additional subxiphoid port (5 mm) for lower CBD or through the umbilical port for upper CBD, was used to fragment the stones by means of intracorporeal contact lithotripsy using a pneumatic lithotripter. The sediments were then cleared by means of forceful saline lavage. Confirmation of CBD clearance was done by post-exploratory cholangiogram in 32 patients and choledochoscopy in 130 patients. Completion choledochoscopy was performed by means of a pediatric bronchoscope (Pentex FB-15P). Following this, a guide wire was then passed through the side channel of the bronchoscope over which a 7 Fr, 10-cm double-flap biliary stent was guided into the CBD up to the duodenum across the sphincter. The CBD was then closed by interrupted 3-0 vicryl sutures.

The cystic duct was then divided and the gallbladder was then lifted up from the liver bed and removed with the extracted stones in an endobag. Hemostasis was secured, a subhepatic drain was placed and the abdomen was deflated.

## RESULTS

In the time period between August 2000 and March 2005, 100 patients underwent antegrade CBD stenting following LCBDE at our institution.

The median duration of the operation was 75 min (55-155) min. Early oral intake and early ambulation was encouraged. Most of the patients were allowed to have liquids on the day of the operation and were ambulatory the next day. The mean postoperative hospital stay was 3.5 days (2-7 days). The intra-abdominal subhepatic drains were removed once the output was less than 20 ml/day. By the third postoperative day, all but one patient had their drains removed. The patients with cholecysto-enteric fistula were allowed oral intake from the third postoperative day.

There was no conversion in the series nor was there any mortality. After discharge, the patients were followed up at 1week, then monthly for 2 months and subsequently were asked to come for annual checkups for 2 years. The stent was removed endoscopically after 4 weeks at the first monthly visit.

One patient had a minor biliary leak via the drainage tube, which stopped spontaneously on the fifth postoperative day and the tube could be removed on the sixth postoperative day. Four patients developed port infection, which was controlled with antibiotics. A male patient who had a cholecystoduodenal fistula reported with fever after 2 weeks. Abdominal ultrasonogram showed a right-sided subhepatic collection, which yielded pus on guided aspiration. The collection was fully aspirated and the patient was admitted and treated conservatively with parenteral antibiotics. He had an otherwise uneventful recovery.

Prolonged ileus (till the fourth postoperative day) associated with hypokalemia was encountered in a 71-year-old diabetic male patient. The ileus resolved on the fifth postoperative day and the patient was discharged the next day.

The stents were removed endoscopically after 4 weeks in all the patients. The follow-up protocol at our institute entails postoperative checkups at the end of the first and the fourth week. Similar protocol of removal of stent at 4 weeks after operation was followed by Isla Griniatos and Wan at Ealing Hospital, London.[7] Selectively, ERCP was performed at stent removal to assess the status of the CBD. One patient had transient cholangitis following stent removal, which was controlled with parenteral antibiotics. All but 17 patients were followed up regularly till the third postoperative month. Follow-up till the first year could be completed in 68 patients. There has been no incidence of residual disease and all the patients who were in regular follow-up have been asymptomatic.

## DISCUSSION

LCBDE is now a frequently performed operation by surgeons experienced in advanced minimal access surgeries. LC with LCBDE could be performed as a single stage procedure in a large series.[8] LCBDE has been shown to be as effective as endoscopic stone removal with lesser procedural time and shorter hospital stay.[9] This allows the preservation of the functions of sphincter of Oddi and thus does away with the risks of cholangitis, pancreatitis and, as a long-term possibility, malignancy, etc, following a sphincterotomy. It has also been shown to be a more cost-effective procedure than endoscopic clearance of the CBD.[10–12]

Following the principles of open surgery, a surgeon has multiple options regarding the management of choledochotomy following LCBDE:

- Primary closure with / without an antegrade biliary stent[8,13–15]
- Closure over a T-tube[15–19]
- Bilio-enteric bypass (in indicated patients)[19,20]

In this study period of LCBDE, it was nearly 2 years before we attempted closure of choledochotomy with an antegrade biliary stent. This happened once we started performing completion choledochoscopy for confirmation of CBD clearance. Post-exploratory cholangiogram was found to be technically cumbersome, with false interpretation due to leaks and air bubbles.

In the initial period, a CBD dilated beyond 1.5 cm was treated with a choledocho-enteric anastomosis, while T-tube drainage was performed in the undilated ones. The presence of a T-tube is associated with a few known disadvantages - patient discomfort, an increased hospital stay, delayed recovery, tube displacement / dislodging, infection and fragmentation, to name a few.[21] This led us to think about offering alternative options to our patients. Our experiences at transcystic exploration were also not encouraging. In two patients in whom we attempted a transcystic CBD exploration, a change of operative approach to choledochotomy was precipitated as we failed to achieve CBD clearance through cystic duct. The ease of direct CBD exploration and our failure with transcystic approaches led us to follow a protocol of laparoscopic CBD exploration as has been described in this article. This is as true for laparoscopic surgery as it is for ‘open’ surgery.[22–24] Hence we decided to try primary closure of the CBD following LCBDE in our patients, our decision being based on available encouraging reports.[8,25–27] Unfortunately, two out of the four patients in whom we attempted primary closure of the CBD developed bilioma in the immediate postoperative period and re-laparoscopy had to be done, where leak was found from the choledochotomy site in both the patients and was subsequently closed over a T-tube. This experience prevented us from attempting any other primary closure of unstented CBD.

Report of an animal study published earlier[28] led us to conceptualize closure of the CBD over an antegrade biliary stent. The first report of laparoscopic primary closure of the CBD had come from Lange V and his team.[29] We had started performing choledochoscopies followed by antegrade biliary stenting by then. The initial experiences (8 patients – 5 female and 3 male) over a 3-month period were encouraging and we persisted with the technique.

Recently, modified plastic biliary stents (after removing the proximal flap) have also been used[30] and there have been more reports describing the success of this procedure,[31,32] which had been already found to be a preferred technique in animal experiments as well.[33] We however do not remove the proximal flap for the fear of premature expulsion of the stent.

The experience of our first hundred patients where an antegrade biliary stent had been used as an adjunct to CBD closure has been encouraging. There has been no conversion or mortality. The incidence and complication has been minimal [Table 5].

**Table 5:** Complication following antegrade stenting after laparoscopic common bile duct exploration

Port infection	4
Minor biliary leak	1
Subhepatic collection	1
Prolonged ileus	1
Cholangitis after stent removal	1
Haematoma (umbilical port)	1
Total	9

In these days of evidence-based incidence, we have instituted a protocol according to which our patients having choledocholithiasis with cholelithiasis are treated. The principles of the protocol include the following:

- Defined indications for preoperative ERCP [Table 1]
- Defined indications for LC with LCBDE [Table 1]
- LCBDE performed via choledochotomy directly. Transcystic LCBDE was avoided because of inherent limitations and possible contraindications / complications [Table 4]
- Choledocho-enteric anastomosis is performed after LCBDE if CBD diameter is >1.5 cm
- All other patients undergo closure of choledochotomy with an antegrade biliary stent, which is removed endoscopically after 4 weeks.

## CONCLUSION

A single stage laparoscopic treatment of cholelithiasis with choledocholithiasis has been found to be safe, viable and cost-effective. Closure of the CBD over an antegrade stent after LCBDE is a feasible option but requires advanced skills in minimal access surgical techniques, especially endosuturing. Endoscopic removal of the stent after a safe interval of 4 weeks does not result in significantly added morbidity and cost of the treatment procedure.
